# Correlation between emotional regulation and peripheral lymphocyte counts in colorectal cancer patients

**DOI:** 10.7717/peerj.9475

**Published:** 2020-07-14

**Authors:** Estela Kakoo Brioso, Sérgio Ferreira Cristina, Luis Costa, Silvia Ouakinin

**Affiliations:** 1 Clínica Universitária de Psiquiatria e Psicologia Médica, Faculdade de Medicina, Universidade de Lisboa, Lisboa, Portugal; 2 Unidade Funcional de Medicina Interna, Hospital de Cascais Dr. José de Almeida, Cascais, Portugal; 3 Serviço de Oncologia Médica, Centro Hospitalar Universitário Lisboa Norte, EPE, Lisboa, Portugal

**Keywords:** Colorectal cancer, NK cells, Anger, Depression, Anxiety, Immune system, T Cells

## Abstract

**Background:**

Colorectal cancer is one of the most common cancers worldwide. Psychological morbidity has an important impact on quality of life and major clinical outcomes. Several data have shown that the immune system may be a key player on the relation between psychological features and cancer outcomes. Natural Killer (NK) cells have been shown to be influenced by psychological factors. The aim of this investigation was to assess the impact of anxiety, depression, and anger state, trait, and expression on the immune response, particularly, their effect on NK cells and CD8^+^ T cells in surgical colorectal cancer patients.

**Methods:**

We studied 54 surgical colorectal cancer patients and assessed patients pre-surgically, post-surgically, and 12 months after surgery (follow-up). We applied the Hospital Anxiety and Depression Scale and the State-Trait Anger Expression Inventory and measured peripheral T cells, CD8^+^ T cells, and NK cells. We did a cross-sectional analysis as well as a longitudinal assessment of the variables during the follow-up period.

**Results:**

Pre-surgical assessment: Trait anger, angry reaction, and anger-out had a significant negative correlation with NK cells. The lymphocytes values were unaffected by the presence of clinical anxiety or depression. Post-surgical assessment: Patients without clinical anxiety had higher levels of T cells. Angry reaction was negatively correlated with NK cells. Lymphocytes values were unaffected by the presence of clinical depression. Follow-up assessment: Patients without clinical depression had higher T cell counts. Trait anger and angry reaction were negatively correlated with the levels of NK cells. The lymphocytes values were unaffected by the presence of clinical anxiety. Longitudinal assessment: Angry-temperament, anger expression, and anger-in reduced significantly from the first to the second assessment. Anxiety, state anger, and trait anger significantly diminished from the pre-surgical to the follow-up assessment. Depression levels did not alter during the follow-up period. The lymphocyte count, and particularly T cells and CD8^+^ T cells, was significantly higher in the follow-up when compared with the pre-surgical assessment.

**Conclusion:**

Our study suggests the existence of a relation between psychological response and immune response in colorectal cancer patients. We identified the importance of emotional regulation as a potential modulator for NK cell counts. Higher values of propensity to experience anger states and express them outwards seem to be associated with lower NK cell counts.

## Introduction

Cancer constitutes a serious public health problem involving elevated costs, both financially and socially. Colorectal cancer is the third most common cancer in men and the second in women worldwide. In 2012, the International Agency for Research on Cancer registered 1.4 million new cases and 694,000 deaths worldwide (Torre et al., 2015).

In oncology, psychiatric disorders, particularly anxiety, depression, and adjustment disorders, are present in 25–30% of patients (Mitchell et al., 2011). Cancer patients have between 14% and 24% more cases of depression than in the general population (Graça Pereira, Figueiredo & Fincham, 2012). In a study conducted with 2,924 cancer patients, 7.8% reported having thoughts that they would be better off dead or thoughts of hurting themselves (Walker et al., 2008). When considering colorectal cancer alone, almost twice the prevalence (14.3%) of patients with persistent suicidal thoughts was found (Shaheen Ahwal et al., 2015).

Psychological morbidity has an important impact on quality of life and major clinical outcomes (Brown et al., 2003). According to the National Comprehensive Cancer Network, all patients with cancer experience some level of distress during their illness and it seems to have a significant compromise of quality of life and survival (NCCN Clinical Practice Guidelines in Oncology Distress Management Version 1, 2018; Spiegel, 2012).

Distress impairs functioning and health behaviors and psychological morbidities, such as depression and anxiety, significantly increase mortality rates and length of hospital stay and worsen clinical outcomes, leading to lower 5-year survival rates than those of patients with no psychological morbidities (Watts et al., 2015; Thekdi, Trinidad & Roth, 2015; Spiegel & Riba, 2015; Watson et al., 1999).

Several data have shown that the immune system may be a key player on the relation between psychological features and cancer outcomes (Green McDonald, O’Connell & Lutgendorf, 2013). Particularly, depression and stress have been associated with changes in immune response (Miller, 2010). Natural Killer (NK) cells have consistently been shown to be influenced by psychological factors (Sachs et al., 1995; Orsi et al., 1996). Steel *et al* have shown an association between depressive symptoms, decreased NK cell numbers and decreased survival in hepatobiliar carcinoma patients (Steel et al., 2007). Also, social support and distress affect cellular immune response both in peripheral blood and at the tumor level (Lutgendorf et al., 2005). The impact of anxiety, dysphoria and anger on the immune system is not only reflected by a decreased cell number but also by an impaired NK cell activity and response to cytokines (Ben-Eliyahu et al., 2000; Irwin, 2002; Fawzy et al., 1990; Tashiro et al., 2001; Levy et al., 1987).

The importance of immune surveillance in colorectal cancer patients is patent in the observation that patients with higher lymphocyte counts also show longer survival (Qiu et al., 2009; Mlecnik et al., 2011). In stage III colorectal cancer, patients with higher pre-surgical levels of lymphocytes associated with cellular immunity (CD3 ^+^, CD4^+^, CD8^+^, and CD16^+^) had longer survival (Milasiene, Stratilatovas & Norkiene, 2007).

In recent studies, tumor-infiltrated lymphocytes, particularly CD8 ^+^ and NK cells, have been shown to constitute a better indicator of tumor recurrence than the American Joint Committee on Cancer (AJCC) / International Union Against Cancer (UICC) - TNM staging (Mlecnik et al., 2011).

The aim of this investigation was to assess the impact of anxiety, depression, and anger state, trait, and expression on the antitumoral immune response, particularly, their effect on NK cells and CD8^+^ T cells in surgical colorectal cancer patients. We postulated that patients with clinical anxiety or depression would present lower lymphocyte counts. Additionally, we hypothesized that specific anger expression patterns would have impact on the immune system and, more specifically, that an anger repression pattern would be associated with fewer circulating lymphocytes.

## Materials & Methods

Data sampling was conducted at the Centro Hospitalar Universitário Lisboa Norte—Hospital de Santa Maria—between January 2012 and July 2015. The hospital ethical committee approved the study and all patients signed an informed consent form.

The participants were patients with a diagnosis of colorectal cancer (adenocarcinoma) undergoing elective surgery.

Patients incapable of comprehending and fulfilling what was required, unaware of their diagnosis, with age below 40, with known metastases at the time of surgery, who underwent neo-adjuvant chemotherapy or radiotherapy or who had high probability of surgical treatment with subsequent colostomy were excluded from the study.

The research was structured in three assessment moments—pre-surgical (24 h period prior to the surgery), post-surgical (one month after surgery), and follow-up (12 months after surgery). We did a cross-sectional analysis of each moment as well as a longitudinal assessment of the variables during the follow-up period.

For evaluation of the psychological variables, we applied the Hospital Anxiety and Depression Scale (HADS) (Zigmond & Snaith, 1983; Pais-Ribeiro et al., 2007), and the State-Trait Anger Expression Inventory (STAXI) (Spielberger, 1988; Silva, Campos & Prazeres, 1999).

The Hospital Anxiety and Depression Scale (HADS) is a 14-item questionnaire, of which seven assess anxiety (HADS-A) and seven assess depression (HADS-D). Each item is scored from 0 to 3, to a maximum of 21 (Zigmond & Snaith, 1983; Pais-Ribeiro et al., 2007). Scores between 0 and 7 are considered non-clinical for both depression and anxiety while scores above 7 in HADS-D or HADS-A represent clinical depression or anxiety respectively. Cronbach’s alpha for the Portuguese version was 0.76 for anxiety and 0.81 for depression (*n* = 1,322).

The State-Trait Anger Expression Inventory (STAXI) is a 44-item inventory, divided into six scales and two subscales, which assess anger experience, expression and control (Spielberger, 1988; Silva, Campos & Prazeres, 1999). STAXI is comprised of (1) State Anger (S-Anger), which measures the intensity of anger feelings at a giving time; (2) Trait Anger (T-Anger) which measures the differences between individuals in the frequency and disposition for experiencing anger states, including two subscales: (a) Angry-temperament (T-Anger/T), which evaluates the general propensity for experiencing and expressing anger feelings with no specific provocation; and (b) Angry Reaction (T-Anger/R) which gauges the differences in anger expression between individuals when criticized or unfairly treated; 3) Anger-in (AX/In), which assesses the frequency with which anger feelings are repressed; 4) Anger-out (AX/ Out), which assesses how frequently anger is directed towards other people and objects; 5) Anger Control (AX/ Con), which assesses the frequency with which the individual tries to control the expression of his/her anger; and 6) Anger Expression (AX/EX), which indicates the frequency with which anger is expressed, regardless of how it is expressed.

Lymphocyte counts in the peripheral blood were obtained by flow cytometry using FACSCalibur^TM^ (Becton Dickinson Biosciences, CA, USA). We used the Anti-CD3 FITC (Becton Dickinson Biosciences, CA, USA), Anti-CD56 PE (Becton Dickinson Biosciences, CA, USA), and the Anti-CD8 APC (Becton Dickinson Biosciences, CA, USA) for selection of T cells (CD3^+^), CD8^+^ T cells (CD3^+^ CD8^+^), and Natural Killer cells (CD3 ^−^ CD56^+^).

Statistical analysis was performed using IBM^®^ SPSS^®^ Statistics version 22. Whenever possible, we used the T-test to compare differences between two independent groups; otherwise the non-parametric Mann-Whitney U test was used. Similarly, when possible, we used the T-test for paired groups and resorted to the Wilcoxon test when the assumptions of a parametric test could not be met. For correlation analysis we used Spearman’s correlation. *p*-values ≤ 0.05 were considered statistically significant. All tests were two-tailed.

## Results

**Figure 1:**
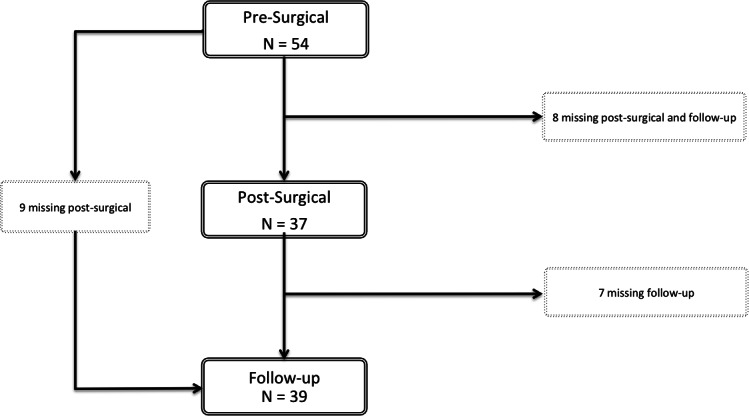
Flowchart of the participants. Fifty-four patients were selected for participation. Of these, nine patients missed the post-surgical assessment but were present for the follow-up evaluation. Eight patients participated only in the pre-surgical assessment and seven patients completed the pre-surgical and post-surgical evaluation, but not the follow-up. Thirty patients completed the three moments of evaluation.

Fifty-four patients were recruited for the first assessment. Most completed the three assessments but some were not available for the post-surgical or the follow-up assessment (Fig. 1). Sociodemographic and clinical data of the participants are represented in Table 1.

**Table 1:** Sociodemographic and clinical characteristics of sample.

	**Pre-surgical**		**Post-surgical**			**Follow-Up**			
	*N* = 54		*N* = 37			*N* = 39			
****	**Mean**	**SD**	**Mean**	**SD**		**Mean**	**SD**		
**Age (years)**	67	10.350	67	9.459		66	8.893		
****	**N**	**%**	**N**	**%**		**N**	**%**		
**Gender**									
Male	32	59.3	23	62.2		25	64.1		
Female	22	40.7	14	37.8		14	35.9		
**Marital Status**									
Married	37	68.5	27	73		29	74.4		
Single, never married	3	5.6	2	5.4		2	5.1		
Separated/divorced	3	5.6	1	2.7		1	2.6		
Widowed	11	20.4	7	18.9		7	17.9		
**Employment**									
Retired	29	53.7	22	59.5		19	48.7		
Employed	14	25.9	7	18.9		10	25.6		
Housewife	9	16.7	6	16.2		8	20.5		
Unemployed	2	3.7	2	5.4		2	5.1		
**Primary Cancer Diagnosis**									
Colon cancer	49	90.7	34	91.9		36	92.3		
Rectal cancer	5	9.3	3	8.1		3	7.7		
**AJCC/UICC-TNM Staging**									
Stage 0	4	7.4	3	8.1		3	7.7		
Stage I	10	18.5	6	16.2		8	20.5		
Stage II	15	27.8	11	29.7		10	25.6		
Stage III	24	44.4	17	45.9		18	46.2		
Stage IV	1	1.9	0	0		0	0		

Pre-surgical assessment: Of the 54 participants, 32 were males and the mean age was 67 years (SD=10.350). Forty-nine patients had colon cancer (90.7%) and 5 patients had rectal cancer. Several patients (42.6%) presented clinical anxiety and 22.2% had clinical depression. Mean values of anxiety, depression, and anger experience, expression and control are depicted in Table 2.

**Table 2:** Anxiety, depression and experience, expression and control of anger.

	**Pre-Surgical**	**Post-Surgical**	**Follow-Up**
****	**Mean ± SD**	**Mean ± SD**	**Mean ± SD**
**Anxiety**	6.93 ± 4.690	5.81 ± 4.768	4.21 ± 3.412
**Depression**	5.50 ± 4.625	5.39 ± 4.765	4.23 ± 3.224
**Anger**			
Anger State	10.72 ± 1.956	10.31 ± 0.951	10.10 ± 0.502
Anger Trait	14.89 ± 3.440	14.03 ± 2.923	13.59 ± 2.479
Angry Temperament	5.80 ± 1.459	5.19 ± 1.546	5.33 ± 1.177
Angry Reaction	6.39 ± 2.087	6.33 ± 1.852	5.79 ± 1.436
Anger-In	15.91 ± 3.773	15.69 ± 2.755	14.85 ± 3.580
Anger-Out	12.09 ± 2.776	11.06 ± 1.820	12.00 ± 2.856
Anger Control	26.20 ± 4.716	27.25 ± 4.211	26.69 ± 4.959
Anger Expression	17.80 ± 7.786	15.50 ± 6.322	16.15 ± 7.707
****	**N (%)**	**N (%)**	**N (%)**
**Anxiety**			
Non Clinical	31 (57.4)	23 (63.9)	32 (82.1)
Clinical	23 (42.6)	13 (36.1)	7 (17.9)
**Depression**			
Non Clinical	42 (77.8)	26 (72.2)	34 (87.2)
Clinical	12 (22.2)	10 (27.8)	5 (12.8)

The pre-surgical mean value of lymphocytes in the peripheral blood was 1.91 × 10^9^/L (SD = 0.742 × 10^9^/L), including 71.7% of T cells, 23.9% of cytotoxic T cells, and 14.5% of NK cells (Table 3). Trait anger was negatively correlated with NK cell counts (*R*=−0.327; *p*-value = 0.018). A negative correlation with NK cells counts was also found for angry reaction (*R* =  − 0.291; *p*-value = 0.036), and anger-out (*R* =  − 0.284; *p*-value = 0.041) (Table 4). The lymphocytes values were unaffected by the presence of clinical anxiety or depression (Table 5).

**Table 3:** Peripheral lymphocytes.

	**Pre-Surgical**	**Post-Surgical**	**Follow-Up**	
****	**Mean ± SD**	**Mean ± SD**	**Mean ± SD**	
**Total Lymphocytes (x 10**^**9**^**/L)**	1.91 ± 0.742	2.08 ± 0.906	2.20 ± 0.601	
**T Cells (x 10**^**9**^**/L)**	1.36 ± 0.546	1.48 ± 0.680	1.59 ± 0.463	
**CD8**^+^**T Cells (x 10**^**9**^**/L)**	0.48 ± 0.396	0.52 ± 0.466	0.59 ± 0.304	
**NK Cells (x 10**^**9**^**/L)**	0.28 ± 0.182	0.31 ± 0.217	0.31 ± 0.185	

**Table 4:** Correlation between psychological variables and peripheral lymphocytes in the pre-surgical assessment.

****	**Total Lymphocytes**	**T Cells**	**CD8**^+^**T Cells**	**NK Cells**
Anger				
Anger State	*R* = 0.141	*R* = 0.213	*R* = 0.083	*R* = 0.044
*p*-value	0.311	0.130	0.577	0.758
Anger Trait	*R* = − 0.045	*R* = 0.066	*R* = 0.055	*R* = − 0.327
*p*-value	0.745	0.643	0.699	0.018^*^
Angry Temperament	*R* = − 0.115	*R* = − 0.051	*R* = − 0.037	*R* = − 0.229
*p*-value	0.407	0.718	0.795	0.102
Angry Reaction	*R* = − 0.020	*R* = 0.074	*R* = 0.056	*R* = − 0.291
*p*-value	0.887	0.600	0.692	0.036^*^
Anger-In	*R* = 0.053	*R* = − 0.010	*R* = 0.043	*R* = − 0.064
*p*-value	0.703	0.945	0.762	0.650
Anger-Out	*R* = − 0.074	*R* = 0.032	*R* = − 0.016	*R* = − 0.284
*p*-value	0.593	0.822	0.913	0.041^*^
Anger Control	*R* = 0.160	*R* = 0.203	*R* = 0.245	*R* = 0.219
*p*-value	0.248	0.148	0.080	0.119
Anger Expression	*R* = − 0.083	*R* = − 0.101	*R* = − 0.129	*R* = − 0.253
*p*-value	0.548	0.474	0.361	0.071
Anxiety	*R* = − 0.048	*R* = − 0.017	*R* = − 0.042	*R* = − 0.052
*p*-value	0.730	0.903	0.769	0.713
Depression	*R* = 0.157	*R* = 0.091	*R* = 0.020	*R* = 0.154
*p*-value	0.257	0.520	0.887	0.274

**Notes.**

* *p*-value ≤ 0.05.

** *p*-value ≤ 0.01.

**Table 5:** Lymphocyte counts according to the presence of clinical anxiety or depression in the pre-surgical assessment.

	Anxiety			Depression		
		Non Clinical	Clinical		Non Clinical	Clinical
		Mean ± SD	Mean ± SD		Mean ± SD	Mean ± SD
Total Lymphocytes (x10^9^/L)		2.01 ± 0.899	1.78 ± 0.440		1.94 ± 0.820	1.80 ± 0.361
*p*-value		0.552			0.617	
T Cells (x10^9^/L)		1.40 ± 0.655	1.31 ± 0.355		1.38 ± 0.599	1.31 ± 0.324
*p*-value		0.941			0.602	
CD8^+^ T Cells (x10^9^/L)		0.53 ± 0.498	0.43 ± 0.180		0.50 ± 0.440	0.45 ± 0.197
*p*-value		0.897			0.664	
NK Cells (x10^9^/L)		0.32 ± 0.207	0.24 ± 0.131		0.28 ± 0.189	0.28 ± 0.164
*p*-value		0.266			0.745	

Post-surgical assessment: Thirty-seven patients participated on the second assessment. Of those, 23 were males. Thirty-four patients had colon cancer (91.9%) and 3 patients had rectal cancer (Table 1). One third of the patients (36.1%) presented clinical anxiety and 27.8% had clinical depression. Mean values of anxiety, depression, and anger experience, expression and control are depicted in Table 2.

The post-surgical mean value of lymphocytes in the peripheral blood was 2.08 × 10^9^/L (SD=0.906 × 10^9^/L), including 72.3% of T cells, 25.0% of cytotoxic T cells, and 14.1% of NK cells (Table 3).

Patients without clinical anxiety had higher levels of T cells compared to the group of patients with clinical anxiety (1.69 × 10 ^9^/L and 1.23 × 10^9^/L, respectively; *p*-value = 0.039) (Table 6). Angry reaction was negatively correlated with NK cells (*R* =  − 0.412; *p*-value = 0.033) (Table 7). Similarly to the pre-surgical assessment, the lymphocytes values were unaffected by the presence of clinical depression (Table 6).

**Table 6:** Lymphocyte counts according to the presence of clinical anxiety or depression in the post-surgical assessment.

	Anxiety			Depression		
		Non Clinical	Clinical		Non Clinical	Clinical
		Mean ± SD	Mean ± SD		Mean ± SD	Mean ± SD
Total Lymphocytes (x10^9^/L)		2.36 ± 1.082	1.76 ± 0.347		2.27 ± 1.058	1.81 ± 0.326
*p*-value		0.080			0.298	
T Cells (x10^9^/L)		1.69 ± 0.800	1.23 ± 0.305		1.60 ± 0.795	1.30 ± 0.276
*p*-value		0.039			0.322	
CD8^+^ T Cells (x10^9^/L)		0.65 ± 0.584	0.37 ± 0.084		0.60 ± 0.560	0.40 ± 0.131
*p*-value		0.342			0.743	
NK Cells (x10^9^/L)		0.36 ± 0.262	0.26 ± 0.129		0.34 ± 0.255	0.27 ± 0.121
*p*-value		0.544			0.743	

**Table 7:** Correlation between psychological variables and peripheral lymphocytes in the post-surgical assessment.

****	**Total Lymphocytes**	**T Cells**	**CD8**^+^**T Cells**	**NK Cells**
Anger				
Anger State	*R* = 0.079	*R* = 0.034	*R* = − 0.089	*R* = 0.159
*p*-value	0.697	0.866	0.659	0.430
Anger Trait	*R* = 0.079	*R* = − 0.056	*R* = − 0.153	*R* = − 0.294
*p*-value	0.696	0.783	0.445	0.137
Angry Temperament	*R* = 0.271	*R* = 0.106	*R* = − 0.320	*R* = − 0.044
*p*-value	0.172	0.598	0.104	0.826
Angry Reaction	*R* = − 0.215	*R* = − 0.260	*R* = − 0.126	*R* = − 0.412
*p*-value	0.282	0.190	0.530	0.033*
Anger-In	*R* = − 0.035	*R* = − 0.169	*R* = − 0.206	*R* = − 0.249
*p*-value	0.864	0.399	0.302	0.210
Anger-Out	*R* = 0.054	*R* = 0.175	*R* = 0.296	*R* = − 0.215
*p*-value	0.790	0.383	0.134	0.282
Anger Control	*R* = − 0.206	*R* = − 0.133	*R* = 0.182	*R* = − 0.134
*p*-value	0.302	0.507	0.363	0.505
Anger Expression	*R* = 0.190	*R* = 0.127	*R* = − 0.135	*R* = − 0.092
*p*-value	0.342	0.528	0.502	0.650
Anxiety	*R* = − 0.110	*R* = − 0.151	*R* = − 0.089	*R* = − 0.045
*p*-value	0.585	0.451	0.660	0.825
Depression	*R* = − 0.124	*R* = − 0.117	*R* = − 0.017	*R* = − 0.270
*p*-value	0.538	0.562	0.932	0.172

**Notes.**

* *p*-value ≤ 0.05.

Follow-up assessment: Thirty-nine patients participated on the third assessment. Of those, 25 were males. Thirty-six patients had colon cancer (92.3%) and 3 patients had rectal cancer (Table 1). Clinical anxiety was present in 17.9% of the patients and clinical depression in 12.8%. Mean values of anxiety, depression, and anger experience, expression and control are depicted in Table 2.

The follow-up mean value of lymphocytes in the peripheral blood was 2.20 x 10^9^/L (SD=0.601), including 72.05% of T cells, 26.15% of cytotoxic T cells, and 13.81% of NK cells (Table 3).

Patients without clinical depression had higher levels of T cells compared to the group of patients with clinical depression (1.63 × 10^9^/L and 1.33 × 10^9^/L, respectively; *p*-value = 0.043) (Table 8). Trait anger (*R* =  − 0.377; *p*-value = 0.018) and angry reaction (*R* =  − 0.330; *p*-value = 0.040) were negatively correlated with the levels of NK cells (Table 9). The lymphocytes values were unaffected by the presence of clinical anxiety (Table 8).

**Table 8:** Lymphocyte counts according to the presence of clinical anxiety or depression in the follow-up assessment.

	Anxiety			Depression		
		Non Clinical	Clinical		Non Clinical	Clinical
		Mean ± SD	Mean ± SD		Mean ± SD	Mean ± SD
Total Lymphocytes (x10^9^/L)		2.24 ± 0.640	2.02 ± 0.348		2.23 ± 0.616	2.04 ± 0.505
*p*-value		0.484			0.492	
T Cells (x10^9^/L)		1.62 ± 0.494	1.43 ± 0.246		1.63 ± 0.442	1.33 ± 0.577
*p*-value		0.440			0.043	
CD8^+^ T Cells (x10^9^/L)		0.61 ± 0.321	0.50 ± 0.208		0.61 ± 0.301	0.45 ± 0.324
*p*-value		0.440			0.257	
NK Cells (x10^9^/L)		0.30 ± 0.196	0.33 ± 0.135		0.31 ± 0.191	0.31 ± 0.157
*p*-value		0.419			0.699	

**Table 9:** Correlation between psychological variables and peripheral lymphocytes in the follow-up assessment.

****	**Total Lymphocytes**	**T Cells**	**CD8**^+^**T Cells**	**NK Cells**
Anger				
Anger State	*R* = − 0.229	*R* = − 0.192	*R* = − 0.245	*R* = − 0.180
*p*-value	0.160	0.241	0.133	0.274
Anger Trait	*R* = − 0.013	*R* = 0.048	*R* = − 0.061	*R* = − 0.377
*p*-value	0.939	0.772	0.712	0.018^*^
Angry Temperament	*R* = − 0.095	*R* = − 0.026	*R* = − 0.026	*R* = − 0.250
*p*-value	0.564	0.876	0.875	0.125
Angry Reaction	*R* = 0.031	*R* = 0.089	*R* = − 0.107	*R* = − 0.330
*p*-value	0.849	0.592	0.517	0.040*
Anger-In	*R* = 0.114	*R* = 0.149	*R* = 0.021	*R* = − 0.148
*p*-value	0.490	0.366	0.900	0.370
Anger-Out	*R* = 0.155	*R* = 0.147	*R* = 0.102	*R* = − 0.019
*p*-value	0.346	0.372	0.537	0.907
Anger Control	*R* = 0.014	*R* = 0.022	*R* = − 0.004	*R* = 0.059
*p*-value	0.930	0.894	0.982	0.719
Anger Expression	*R* = 0.003	*R* = 0.010	*R* = − 0.091	*R* = − 0.175
*p*-value	0.986	0.954	0.580	0.287
Anxiety	*R* = − 0.083	*R* = − 0.092	*R* = 0.015	*R* = 0.138
*p*-value	0.616	0.576	0.929	0.401
Depression	*R* = − 0.113	*R* = − 0.196	*R* = − 0.178	*R* = 0.135
*p*-value	0.492	0.231	0.277	0.414

**Notes.**

* *p*-value ≤ 0.05.

** *p*-value ≤ 0.01.

Longitudinal assessment: Angry-temperament, anger expression, and anger-in were significantly reduced on the second assessment when compared to the first assessment (5.75 to 5.19, *p*-value = 0.044; 17.78 to 15.5, *p*-value = 0.049; 16.97 to 15.69, *p*-value = 0.046; respectively). Additionally, anxiety, state anger, and trait anger significantly diminished from the pre-surgical to the follow-up assessment (6.62 to 4.21, *p*-value= 0.001; 10.85 to 10.10, *p*-value = 0.031; 14.62 to 13.59, *p*-value = 0.009; respectively). We did not find significant differences in mean values of anxiety (*p*-value = 0.085), state anger (*p*-value = 0.123), trait anger (*p*-value = 0.336), angry reaction (*p*-value = 0.590), anger-out (*p*-value = 0.126), or anger control (*p*-value = 0.628) between the pre-surgical and post-surgical assessment. When we compared the pre-surgical assessment with the follow-up, we did not find significant differences in mean values of angry temperament (*p*-value = 0.062), angry reaction (*p*-value = 0.060), anger-in (*p*-value = 0.059), anger-out (*p*-value = 0.591), anger control (*p*-value = 0.628), or anger expression (*p*-value = 0.062). No differences were found in mean values of anxiety (*p*-value = 0.182), state anger (*p*-value = 0.492), trait anger (*p*-value = 0.622), angry temperament (*p*-value = 0.094), angry reaction (*p*-value = 0.119), anger-in (*p*-value = 0.797), anger-out (*p*-value = 0.101), anger control (*p*-value = 0.375), or anger expression (*p*-value = 0.418) between the post-surgical and follow-up assessments.

The levels of depression did not alter during the follow-up period. Differences in mean values of depression between the pre-surgical and the post-surgical assessment (*p*-value = 0.882), the pre-surgical and the follow-up assessment (*p*-value = 0.390), and the post-surgical and follow-up assessment (*p*-value = 0.234) were non significant.

The lymphocyte count (2.20 × 10^9^/L to 1.96 × 10 ^9^/L, *p*-value = 0.005), and particularly the T cell (1.40 × 10^9^/L to 1.58 × 10^9^/L, *p*-value = 0.010) and CD8 ^+^ T cell (0.51 × 10^9^/L to 0.58 × 10^9^/L, *p*-value = 0.003) counts, was significantly higher in the follow-up when compared with the pre-surgical assessment. No other changes in lymphocytes were seen during the follow-up.

## Discussion

We observed a reduction in the number of patients with clinical anxiety between the pre-surgical (42.6%) and the follow-up (17.9%) assessment. On the other hand, mean depression levels remained fairly constant at non-clinical values throughout the follow-up period. Bullen et al also found non-clinical mean levels of depression and anxiety in pre-surgical colorectal cancer patients (Bullen et al., 2012).

Depression in cancer patients may be related to the disease itself or with previous experiences. In depressed patients, worries, sadness, and a difficulty in self projection in the future in a positive way seems to be present throughout the disease course, while anxiety seems to be higher when fear and concern are most intense, as in the perisurgical period and during the prospect of initiation of chemotherapy, and lower when farther from surgery.

From the pre-surgical to the post-surgical assessment, there were significant reductions in angry temperament, anger expression, and anger-in. With these results we postulate that, before surgery, patients are more prone to angry feeling without a specific trigger because the experience of receiving a cancer diagnosis has the potential to create angry feeling in itself. As far as the expression of anger is concerned, patients appear to increasingly direct anger outwards instead of directing it inwards during the disease course. In fact, many patients have difficulty in expressing their feelings when in intense stress situations and a significant benefit in interventions aimed at improving emotional expression has been described (Carmack et al., 2011).

There was also a reduction in anger state and anger trait from the pre-surgical to the follow-up assessment. This reduction in the frequency and intensity of the perception of situations as unpleasant or frustrating might be in relation with the fact that, at first, patients go through a period of greater uncertainty and fear related to the disease and its treatments but, after a whole year, they have again regained the perception of being able to control their lives and returning to daily routines and work. This period of less uncertainty and the regaining of health related quality of life might explain the lower anger values found, be it more fleeting (anger state) or more frequent and intense (anger trait).

To the extent of our knowledge, this is the first study describing anger evolution in colorectal cancer patients and, as such, we are unable to compare our results with published evidence and draw any further conclusions.

In the follow-up assessment, clinical depression seems to be associated with lower T cell counts. In contrast, anxiety might have a small influence in T cell counts. Post-surgical patients with clinical anxiety had lower T cell counts than patients without clinical anxiety. However, both groups had mean values similar to the mean value of anxiety of the whole post-surgical sample and high standard deviation, and thus we cannot state that this difference has any real clinical value with the current sample.

In relation to anger evaluation, the disposition and frequency for experiencing anger states (anger trait) showed a significant inverse correlation with NK cell counts both at the pre-surgical and follow-up assessment, indicating a possible influence of anger in innate immunity. The anger trait factor most consistently associated with NK cell counts was the expression of anger when wronged or criticized (angry reaction). As a matter of fact, higher values of angry reaction were associated with lower NK cell counts in every assessment throughout the study.

Anger manifestation towards other people and objects (anger-out) was also identified has having influence in the immune system. Higher levels of anger-out were associated with lower lymphocyte counts in the pre-surgical assessment. Particularly, NK cell counts were lower in patients with higher anger-out levels, again suggesting an impact in the innate immune response.

Overall, in our research, depression and anxiety did not seem to have a clear-cut influence in the studied cellular immune component. However, we found consistent data suggesting the impact of one of the most important factors for psychological wellbeing –anger - in NK cell counts. The disposition for experiencing anger states and, even more important, the propensity to their expression when criticized or wronged seem to have an important relation with NK cell counts and, thus, in the future it might be of interest to identify these anger expression patterns and pursue a therapeutical approach in these patients, particularly if experimental larger scale studies are able to find a benefit in outcomes with intervention.

Despite pointing to a significant effect of the experience and manifestation of anger in circulating lymphocytes, the observational design of the study does not allow us to draw conclusions about the benefit of intervention directed at emotional regulation. In addition, although there were lower NK cell counts in patients with more experience and manifestation of anger, this study did not assess other aspects of the immune response besides circulating lymphocyte counts. The antitumoral immune response is a complex process for which there is a contribution of not only the number of immune cells but also their activity. Therefore, it would be interesting to study if these psychological variables also have a qualitative impact in the immune system beyond the quantitative already observed in our study. The antitumoral response is also dependent on the local tumor immune niche. Several studies have shown the important role of tumor-infiltrated lymphocytes in defining prognosis in these patients but, taking into account that our research only included circulating lymphocytes, it remains to be clarified if the psychological response has any impact on the tumor immune microenvironment (Waldhauer & Steinle, 2008; Mlecnik et al., 2011; Funada et al., 2003).

In our research, we only assessed some lymphocyte populations, however the immune response encompasses a complex network of cellular and protein effectors and, thus, it would be relevant to replicate this study with the inclusion of different immune variables to fully understand how emotional regulation and psychiatric comorbidities might impact the antitumoral immune response in colorectal cancer patients.

We were not able to find an important impact of anxiety and depression on circulating lymphocytes. However, we cannot rule out that the impact on the immune system is only visible in patients with severe depression or anxiety. Also, the small sample size and therefore the small number of patients with severe anxiety or depression did not guarantee enough statistical power to confidently claim that anxiety and depression, regardless of severity, does not impact circulating lymphocyte counts in colorectal patients. It would be important to replicate this study with a larger sample and a longer follow-up time to have a subgroup analyses to answer this question.

Our research is innovative as it is the only one that studies lymphocyte counts in surgical colorectal cancer patients in the Portuguese population. Besides, to the extent of our knowledge, it is the first time that a comprehensive psychological assessment is performed on these patients, as well as the identification of the impact of psychological response in the immune system of colorectal cancer patients. As such, our investigation fills an existing gap in the knowledge of the psychological functioning of Portuguese patients in whom, so far, psychological interventions have been based on data extrapolated from other populations.

Our results seem to point to a significant overall impact of one of the six human basic emotions—anger—on NK cell counts in colorectal cancer patients. We therefore consider of outmost importance the inclusion of anger assessment in multicentric prospective studies to evaluate the impact of this emotion, not only on the immune system, but also on major clinical outcomes in colorectal cancer.

## Conclusions

Our study suggests the existence of a relation between psychological response and cellular immune response in colorectal cancer patients. We identified the importance of emotional regulation, and specifically of anger, as a potential modulator for NK cell counts. Higher values of propensity to experience anger states and express them outwards seem to be associated with lower NK cell counts. We did not recognize a significant impact of depression or anxiety in circulating lymphocytes throughout the follow-up. Additional studies are needed, particularly with a more comprehensive assessment of the immune system including tumor-infiltrated lymphocytes and a larger sample size and longer follow-up time, in order to achieve more robust conclusions. Further investigation should include experimental studies to determine the clinical impact of interventions aiming emotional regulation on the antitumoral immune response and clinical outcomes.

##  Supplemental Information

10.7717/peerj.9475/supp-1 Supplemental Information 1 Raw Data: immune and clinical variables of the participants and encompasses all data used for statistical analysis

## Additional Information and Declarations

### Data Availability

The following information was supplied regarding data availability: The raw data are available as a Supplementary File.

## References

[ref-2] Ben-Eliyahu S, Shakhar G, Page GG, Stefanski V, Shakhar K (2000). Suppression of NK cell activity and of resistance to metastasis by stress: a role for adrenal catecholamines and beta-adrenoceptors. Neuroimmunomodulation.

[ref-3] Brown KW, Levy AR, Rosberger Z, Edgar L (2003). Psychological distress and cancer survival: a follow-up 10 years after diagnosis. Psychosomatic Medicine.

[ref-4] Bullen TL, Sharpe L, Lawsin C, Patel DC, Clarke S, Bokey L (2012). Body image as a predictor of psychopathology in surgical patients with colorectal disease. Journal of Psychosomatic Research.

[ref-5] Carmack CL, Basen-Engquist K, Yuan Y, Greisinger A, Rodriguez-Bigas M, Wolff RA, Barker T, Baum G, Pennebaker JW (2011). Feasibility of an expressive-disclosure group intervention for post-treatment colorectal cancer patients: results of the Healthy Expressions study. Cancer.

[ref-6] Fawzy FI, Kemeny ME, Fawzy NW, Elashoff R, Morton D, Cousins N, Fahey JL (1990). A structured psychiatric intervention for cancer patients. II. Changes over time in immunological measures. Archives of General Psychiatry.

[ref-7] Funada Y, Noguchi T, Kikuchi R, Takeno S, Uchida Y, Gabbert HE (2003). Prognostic significance of CD8+ T cell and macrophage peritumoral infiltration in colorectal cancer. Oncology Reports.

[ref-19] Graça Pereira M, Figueiredo AP, Fincham FD (2012). Anxiety, depression, traumatic stress and quality of life in colorectal cancer after different treatments: a study with Portuguese patients and their partners. European Journal of Oncology Nursing.

[ref-11] Green McDonald P, O’Connell M, Lutgendorf SK (2013). Psychoneuroimmunology and cancer: a decade of discovery, paradigm shifts, and methodological innovations. Brain, Behavior, and Immunity.

[ref-8] Irwin M (2002). Psychoneuroimmunology of depression: clinical implications. Brain, Behavior, and Immunity.

[ref-9] Levy S, Herberman R, Lippman M, d’Angelo T (1987). Correlation of stress factors with sustained depression of natural killer cell activity and predicted prognosis in patients with breast cancer. Journal of Clinical Oncology.

[ref-10] Lutgendorf SK, Sood AK, Anderson B, McGinn S, Maiseri H, Dao M, Sorosky JI, Geest KD, Ritchie J, Lubaroff DM (2005). Social support, psychological distress, and natural killer cell activity in ovarian cancer. Journal of Clinical Oncology.

[ref-12] Milasiene V, Stratilatovas E, Norkiene V (2007). The importance of T-lymphocyte subsets on overall survival of colorectal and gastric cancer patients. Medicina.

[ref-13] Miller AH (2010). Depression and immunity: a role for T cells?. Brain, Behavior, and Immunity.

[ref-14] Mitchell AJ, Chan M, Bhatti H, Halton M, Grassi L, Johansen C (2011). Prevalence of depression, anxiety, and adjustment disorder in oncological, haematological and paliative–care settings: a meta-analises of 94 interview based studies. The Lancet Oncology.

[ref-15] Mlecnik B, Tosolini M, Kirilovsky A, Berger A, Bindea G, Meatchi T, Bruneval P, Trajanoski Z, Fridman W, Pagés F, Galon J (2011). Histopathologic-based prognostic factos of colorectal cancers are associated with the state of the local immune reaction. Journal of Clinical Oncology.

[ref-16] NCCN Clinical Practice Guidelines in Oncology Distress Management Version 1 (2018). National Comprehensive Cancer Network; c2018 Acedido a 01/04/2018. Disponível em.

[ref-17] Orsi AJ, McCorkle R, Tax AW, Barsevick A (1996). The relationship between depressive symptoms and immune status phenotypes in patients undergoing surgery for colorectal cancer. Psychooncology.

[ref-18] Pais-Ribeiro J, Silva I, Ferreira T, Martins A, Meneses R, Baltar M (2007). Validation study of a Portuguese version of the hospital anxiety and depression scale. Psychology, Health & Medicine.

[ref-20] Qiu H, Xiao-Jun W, Zhi-Wei Z, Gong C, Guo-Qiang W, Li-Yi Z, al et (2009). The prognostic significance of peripheral T-lymphocyte subsets and natural killer cells in patients with colorectal cancer. Hepato-gastroenterology.

[ref-21] Sachs G, Rasoul-Rockenschaub S, Aschauer H, Spiess K, Göber I, Staffen A, Zielinski C (1995). Lytic effector cell activity and major depressive disorder in patients with breast cancer: a prospective study. Journal of Neuroimmunology.

[ref-22] Silva DR, Campos R, Prazeres N (1999). O inventário de estado-traço de raiva (STAXI) e a sua adaptação para a população portuguesa. Revista Portuguesa de Psicologia.

[ref-23] Spiegel D (2012). Mind matters in cancer survival. Psychooncology.

[ref-24] Spiegel D, Riba MB (2015). Managing anxiety and depression during treatment. The Breast Journal.

[ref-25] Spielberger CD (1988). State-trait anger expression inventory: professional manual.

[ref-1] Shaheen Al Ahwal MS, Zaben FA, Khalifa DA, Sehlo MG, Ahmad RG, Koenig HG (2015). Depression in patients with colorectal cancer in Saudi Arabia. Psychooncology.

[ref-26] Steel JL, Geller DA, Gamblin TC, Olek MC, Carr BI (2007). Depression, immunity, and survival in patients with hepatobiliary carcinoma. Journal of Clinical Oncology.

[ref-27] Tashiro M, Itoh M, Kubota K, Kumano H, Masud MM, Moser E, Arai H, Sasaki H (2001). Relationship between trait anxiety, brain activity and natural killer cell activity in cancer patients: a preliminary PET study. Psychooncology.

[ref-28] Thekdi SM, Trinidad A, Roth A (2015). Psychopharmacology in cancer. Current Psychiatry Reports.

[ref-29] Torre LA, Bray F, Siegel RL, Ferlay J, Lortet-Tieulent J, Jemal A (2015). Global cancer statistics, 2012. CA: A Cancer Journal for Clinicians.

[ref-30] Waldhauer I, Steinle A (2008). NK cells and cancer immunosurveillance. Oncogene.

[ref-31] Walker J, Waters RA, Murray G, Swanson H, Hibberd CJ, Rush RW, al et (2008). Better off dead: suicidal thoughts in cancer patients. Journal of Clinical Oncology.

[ref-32] Watson M, Havil JS, Greer S, Davidson J, Bliss JM (1999). Influence of psychological response on survival in breast cancer: a population-based cohort study. Lancet.

[ref-33] Watts S, Prescott P, Mason J, McLeod N, Lewith G (2015). Depression and anxiety in ovarian cancer: a systematic review and meta-analysis of prevalence rates. BMJ Open.

[ref-34] Zigmond AS, Snaith RP (1983). The hospital anxiety and depression scale. Acta Psychiatrica Scandinavica.

